# Short-Term Outcomes of Cardiogenic Shock Treated With a Microaxial Flow Pump

**DOI:** 10.1016/j.jacasi.2025.09.011

**Published:** 2025-12-02

**Authors:** Shunsuke Matsushita, Yuichi Sawayama, Kohei Osakada, Shunsuke Kubo, Yuichi Kawase, Takeshi Tada, Yasushi Fuku, Hiroyuki Tanaka, Kazushige Kadota, the J-PVAD Investigators

**Affiliations:** Department of Cardiovascular Medicine, Kurashiki Central Hospital, Kurashiki, Japan

**Keywords:** door-to-unload time, mechanical circulatory support, microaxial flow pump, NSTE-ACS, STEMI

The prognosis of acute coronary syndrome (ACS) has improved with reperfusion therapy advancements.^1^ However, patients presenting with cardiogenic shock (CS) continue to achieve poor outcomes, with in-hospital mortality rates of 40%-50%.^1^^,^^2^ Mechanical circulatory support devices, such as the microaxial flow pump (mAFP) (Impella, Abiomed), provide circulatory stability for patients with CS and are increasingly used in clinical practice.^3^ Robust clinical evidence was limited until recently, but the DanGer Shock (Danish–German Cardiogenic Shock) trial demonstrated mortality reduction with mAFP, clarifying its indications.^4^

Patients with non–ST-segment elevation acute coronary syndrome (NSTE-ACS) generally exhibit distinct pathophysiological characteristics from those with ST-segment elevation myocardial infarction (STEMI).^5^ Short-term prognosis varies in NSTE-ACS across studies,^2^^,^^6^, ^7^, ^8^ whereas mortality rates are consistently lower than in patients with STEMI.^9^ However, mortality rates remain alarmingly high in NSTE-ACS complicated by CS and may even exceed those of patients with STEMI with CS.^2^ Although mAFP is used for NSTE-ACS with CS, large-scale outcome data remain lacking.

## Methods

We conducted a post hoc analysis using data from the J-PVAD (Japanese Registry for Percutaneous Ventricular Assist Devices), a nationwide registry of consecutive patients receiving mAFP in Japan. The study included patients treated for ACS and CS between February 1, 2020 and December 31, 2022. The study was approved by the Central Institutional Review Board (Osaka University, #17,232) and complied with the Declaration of Helsinki. The registry included 2,629 patients with ACS, of whom 2,545 patients met the inclusion criteria, including successful mAFP device insertion, identified ACS type, and available survival data. Patients were categorized into STEMI and NSTE-ACS groups. NSTE-ACS includes both non-STEMI and unstable angina. CS was defined as: 1) prolonged hypotension (systolic blood pressure [SBP] <90 mm Hg, the use of vasoactive inotropes to maintain SBP ≥90 mm Hg, or a ≥30-mm Hg decrease in SBP from baseline); and 2) signs of end-organ hypoperfusion. The primary endpoint was 30-day mortality after device implantation. Secondary outcomes included mAFP-related complications.

Adjusted HRs with 95% CIs were calculated with the Cox proportional hazard regression model. We included the following variables in the multivariable model: ACS type; age; sex; dyslipidemia; diabetes mellitus; prior ischemic stroke or transient ischemic attack; SBP at admission; out-of-hospital cardiac arrest; estimated glomerular filtration rate; simultaneous use of mAFP and extracorporeal membrane oxygenation (ECPELLA); door-to-unload time; and inotrope or vasopressor administration at mAFP placement.

## Results

**Figure 1:**
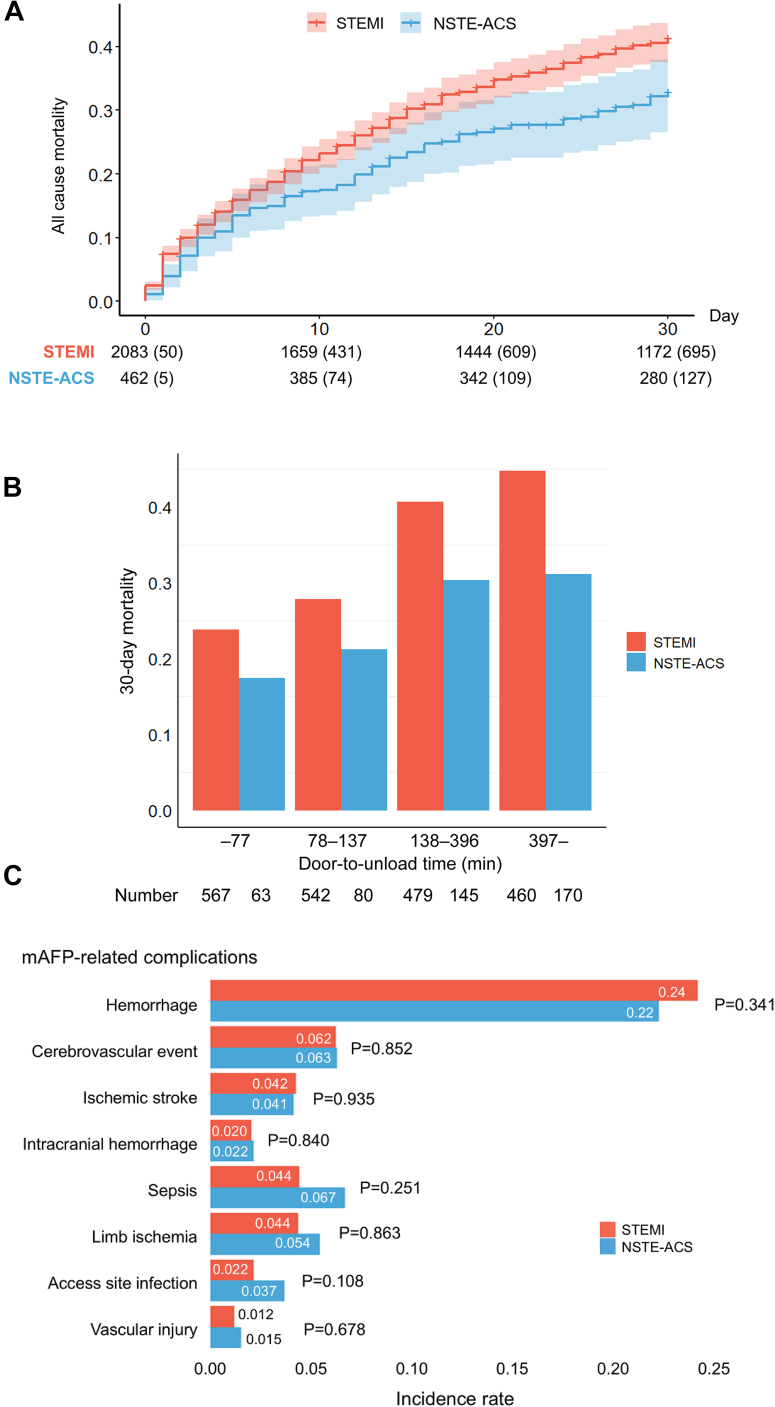
30-Day Survival and In-Hospital Complications by ACS Type (A) Cumulative incidence of 30-day all-cause mortality. Thirty-day mortality was significantly higher in ST-segment elevation myocardial infarction (STEMI) patients than in those with non–ST-segment elevation acute coronary syndrome (NSTE-ACS) (32% vs 26%; HR: 1.46; 95% CI: 1.17-1.81; *P* < 0.001). (B) Thirty-day mortality stratified by quartiles of door-to-unload time. A shorter door-to-unload time was associated with improved survival, irrespective of ACS type. (C) In-hospital complications. Sepsis occurred more frequently in NSTE-ACS patients, whereas the incidence of other complications did not differ significantly between groups. mAFP = microaxial flow pump.

This study included 2,545 patients, with a mean age of 70 ± 12 years. Of the patients, 80% were male and 82% presented with STEMI (n = 2,083). Patients in the NSTE-ACS group were older than those in the STEMI group. Furthermore, the NSTE-ACS group exhibited a higher prevalence of atherosclerotic risk factors (Table 1). Regarding clinical presentation, patients with STEMI demonstrated lower SBP and higher lactate levels at admission. The utilization rate of inotropes and vasopressors at the initiation of mAFP was higher in the STEMI group (*P* < 0.001). Percutaneous coronary intervention was performed more frequently in STEMI, whereas coronary artery bypass graft was more common in NSTE-ACS. The duration of mAFP support was significantly longer in the STEMI group. Moreover, the median door-to-unload time was significantly shorter in the STEMI group. Treatment by ECPELLA was more frequent in the STEMI group than in the NSTE-ACS group. Figure 1A shows significantly higher 30-day mortality in STEMI than in NSTE-ACS (32% vs 26%, log-rank *P* = 0.01). STEMI was also independently associated with increased mortality in multivariable Cox analysis (HR: 1.46; 95% CI: 1.17-1.81; *P* < 0.001). When dividing into quartiles based on the door-to-unload time, the 30-day mortality was lower with shorter door-to-unload time, irrespective of ACS type (both *P* for trend < 0.05) (Figure 1B). Hemorrhage was the most prevalent complication (24%, n = 607), occurring at similar rates between groups (*P* = 0.34). Sepsis was reported in 123 patients (4.8%), with a lower incidence in the STEMI group (STEMI: 4.4% vs NSTE-ACS: 6.7%), although not statistically significant (Figure 1C).

**Table 1:** Patient Characteristics and In-Hospital Management Based on Acute Coronary Syndrome Types

	N	Overall (N = 2,545)	STEMI (n = 2,083)	NSTE-ACS (n = 462)	*P* Value
Age, y	2,545	72 (62-78)	71 (61-78)	74 (67-80)	<0.001
Male	2,545	2,031 (80)	1,674 (80)	357 (77)	0.134
Body mass index, kg/m^2^	2,418	23.5 (21.1-26.1)	23.5 (21.2-26.1)	23.5 (20.6-26.0)	0.498
Current smoker	2,209	802 (36)	683 (38)	119 (29)	0.001
Hypertension	2,422	1,652 (68)	1,298 (66)	354 (79)	<0.001
Dyslipidemia	2,428	1,261 (52)	999 (51)	262 (58)	0.003
Diabetes mellitus	2,457	1,084 (44)	832 (41)	252 (56)	<0.001
Hemodialysis	2,417	101 (4.2)	53 (2.7)	48 (11)	<0.001
Systolic blood pressure, mm Hg	2,545	93 (76-114)	92 (75-112)	98 (80-121)	<0.001
Heart rate, beats/min	2,545	90 (73-110)	90 (73-110)	90 (75-109)	0.970
Lactate, mmol/ L	1,876	4.2 (2.3-8.1)	4.4 (2.4-8.2)	3.6 (1.8-7.5)	<0.001
LVEF, %	1,289	30 (20-40)	30 (20-40)	30 (20-42)	0.238
OHCA	2,531	368 (15)	325 (16)	43 (9.4)	<0.001
Revascularization					
PCI	2,545	2,302 (90)	1,904 (91)	398 (86)	<0.001
CABG	2,545	239 (9.4)	158 (7.6)	81 (18)	<0.001
mAFP device	2,545				0.700
Impella 2.5		87 (3.4)	70 (3.4)	17 (3.7)	
Impella CP		2,380 (94)	1,952 (94)	428 (93)	
Impella 5.0		60 (2.4)	46 (2.2)	14 (3.0)	
Impella 5.5		18 (0.7)	15 (0.7)	3 (0.6)	
Duration of mAFP, day	2,542	3.6 (1.7-6.7)	3.7 (1.8-6.7)	3.0 (1.0-6.3)	0.004
Door-to-unload time, min	2,506	137 (77-396)	122 (74-308)	247 (115-1,297)	<0.001
mAFP before PCI	1,527	1,226 (80)	997 (80)	229 (83)	0.169
Successful weaning	2,545	1,680 (66)	1,360 (65)	320 (69)	0.103
ECPELLA	2,545	919 (36)	772 (37)	147 (32)	0.034

Values are median (Q1-Q3) or n (%). A 2-sided *P* value of <0.05 indicated statistical significance. N represents the total number of valid data available for the entire population.

CABG = coronary artery bypass graft; ECPELLA = simultaneous use of mAFP and extracorporeal membrane oxygenation; LVEF = left ventricular ejection fraction; mAFP = microaxial flow pump; NSTE-ACS = non–ST-segment elevation acute coronary syndrome; OHCA = out-of-hospital cardiac arrest; PCI = percutaneous coronary intervention; STEMI = ST-segment elevation myocardial infarction.

## Discussion

This nationwide registry analysis revealed that 30-day mortality was significantly higher in the STEMI group, and a shorter door-to-unload time was associated with improved survival. This time-mortality association remained significant in multivariable analysis for STEMI, but not for NSTE-ACS, likely due to the smaller sample size and different pathophysiological dynamics in the latter. These results suggest that early mAFP support is particularly critical in STEMI-related CS, and that prognostic factors may differ by ACS subtype.

The higher 30-day mortality in STEMI patients, despite more favorable risk profiles and shorter door-to-unload times, likely reflects the distinct pathophysiology of STEMI-related CS. In STEMI, acute coronary artery occlusion or critical flow limitation often causes rapid and extensive myocardial ischemia, leading to larger infarct size and more severe metabolic derangements.^6^^,^^10^ In our cohort, STEMI patients exhibited significantly higher lactate levels at presentation, suggesting profound tissue hypoperfusion and systemic stress. Even with timely revascularization and mechanical circulatory support, irreversible myocardial injury and inflammatory responses may precipitate multiorgan dysfunction in STEMI. Furthermore, STEMI may involve mechanical complications (eg, ventricular septal rupture), which further worsen outcomes.^10^ By contrast, NSTE-ACS is commonly associated with subtotal occlusion or fluctuating ischemia, allowing for more gradual hemodynamic deterioration and better tolerance of delayed support initiation. These differences in ischemic burden and shock dynamics likely contribute to the mortality gap between ACS subtypes.

This study has several limitations. This is a retrospective registry analysis, which is subject to selection bias and residual confounding. The lack of detailed procedural data, such as door-to-balloon time, use of rotational atherectomy, left main trunk bifurcation stenting, thrombosuction, and glycoprotein IIb/IIIa inhibitors, is an inherent limitation of the registry. Long-term outcomes and data on infarct-related artery or left ventricular ejection fraction were unavailable. Results may be influenced by institutional differences in protocols and operator experience. Quartile distribution was skewed in NSTE-ACS, limiting interpretation of time-mortality association. Nonetheless, this study provides meaningful insights into the differential characteristics and time-sensitive nature of mAFP therapy across ACS subtypes, reinforcing the need for tailored management strategies in CS.

## Financial Support and Author Disclosures

The authors have reported that they have no relationships relevant to the contents of this paper to disclose.
